# Examination of the gait pattern based on adjusting and resulting components of the stride-to-stride variability: proof of concept

**DOI:** 10.1186/s13104-017-2623-8

**Published:** 2017-07-20

**Authors:** U. Laessoe, N. M. B. Jensen, P. Madeleine

**Affiliations:** 1 Physiotherapy Department, University College of Northern Denmark, UCN, Selma Lagerloeffsvej 2, 9220 Aalborg, Denmark; 2 Department of Research and Development, UCN, Selma Lagerloeffsvej 2, 9220 Aalborg, Denmark; 3 Physical Activity and Human Performance Group-SMI, Department Health Science and Technology, Aalborg University, Fredrik Bajersvej 7D, 9220 Aalborg, Denmark

**Keywords:** Gait variability, Motor control, Locomotion, Gait assessment, Variance evaluation

## Abstract

**Background:**

Stride-to-stride variability may be used as an indicator in the assessment of gait performance, but the evaluation of this parameter is not trivial. In the gait pattern, a deviation in one stride must be corrected within the next strides (elemental variables) to ensure a steady gait (performance variable). The variance in these elemental and performance variables may therefore be evaluated as *adjusting* and *resulting* components of variability. We explored this approach to gait evaluation by matching the velocity of one stride to a subsequent stride with four different time lags ranging from 0.5 to 2 strides with 0.5 stride increments. The time lag values corresponded to the following contralateral stride, the following ipsilateral stride, the second following contralateral stride and the second following ipsilateral stride.

**Methods:**

Twenty asymptomatic young adults walked on an instrumented treadmill at their preferred gait speed. The stride velocity was calculated, and variances in the stride-to-stride differences and in the stride-to-stride sums represented the *adjusting* and the *resulting* variances, respectively. A ratio between these values of greater than one indicated a meaningful stride-to-stride interaction.

**Results:**

For the four time lags (0.5, 1, 1.5, and 2 strides), the *adjusting/resulting* variance ratios (mean and CI 95%) were 1.0 (0.8–1.2), 2.9 (2.3–3.6), 1.2 (1.0–1.4) and 1.2 (0.9–1.4), respectively.

**Conclusions:**

This new approach to the evaluation of stride-to-stride variability suggests that gait velocity adjustments occurred within one full stride cycle during treadmill walking among asymptomatic young adults. The validity of the approach needs to be tested in over-ground walking.

## Background

### Gait assessment

Assessing the spatio-temporal aspects of the gait pattern is relevant to the evaluation of human motor performance. The gait characteristics may be derived objectively from spatial or temporal parameters such as gait velocity, step length, step time or double support time, but a summarization of time series may result in the loss of valuable information.

During a clinical examination of the gait, the lack of a steady rhythm in the gait pattern will draw the attention of the clinician. Such an observation will often be interpreted as a deficit in the motor planning or in the postural control of the patient. Accordingly, several studies have observed that evaluation of stride variability may be important when characterizing the gait pattern [1, 2].

### Gait variability

Gait variability has been addressed using a variety of means and methods [2, 3]. The variability of the gait pattern, based on a *discrete time series analysis* of a large number of gait cycles, has been proven to be significant and may reveal information about the maturation of gait function in children [4]. Additionally, more advanced statistics have been used to analyse gait variability, including linear techniques such as the autocorrelation function [5] and non-linear approaches [6, 7] such as approximate entropy [8], sample entropy, the maximum Lyapunov exponent [9] or the detrended fluctuation analysis [10]. However, in a daily clinical context, the gait pattern is most often evaluated by less sophisticated methods.

### Interpretation of variability

An emerging perspective indicates that variability may be used to characterize the level of motor performance [11]. An increase in the stride-to-stride time variability has been associated with fall risk in the elderly [12]. Additionally, challenging dual-task situations and physical impairment may result in greater gait variability [13, 14]. Gait variability is therefore often regarded as an indicator of motor deficits. However, by assessing the complexity (the degree of irregularity using approximate or sample entropy) of a kinematic time series relative to pathology or impaired motor control, i.e., structural variability, both increased and decreased variability of movement characteristics have been reported [11, 15–17].

A given motor task can be performed using different combinations of movements owing to the redundancy of the motor system [18]. When a motor task is repeated, the two actions will never be identical because a certain degree of variation in movement synergies is considered natural [19]. Inherent biomechanical and neuro-motor redundancies are available within the context of the control processes involved [20], and these must also be considered when interpreting the gait pattern.

### Elemental and performance variables in gait

According to a definition by Guthrie [21], skill “consists in the ability to bring about some end result with maximum certainty and minimum outlay of energy, or of time and energy”. A skilled movement strategy, when walking on a flat surface, would result in a steady gait with only natural intrinsic deviations [22]. In terms of energy consumption, the whole-body momentum during walking should be preserved [23]. A change in velocity implies that accelerations, according to Newton’s second law, require force and energy. Therefore, deviations in overall gait velocity or direction are not considered optimal [24].

From a clinical point of view, any tendency toward deviation in the gait pattern during one stride should be corrected at some point within the following strides. These ongoing corrections should be coordinated to ensure a steady gait. With this understanding, the gait performance is a result of the interaction of stride subsystems acting in synergy. A certain degree of stride-to-stride variation may be expected and may be a sign of good motor performance. Therefore, the interpretation of gait variability as an outcome measure is not trivial and subject to further scientific attention [25].

Latash and colleagues have been studying synergies in other settings, and their work with performance and elemental variables may provide inspiration for a new perspective on the variability of the gait pattern [19]. We propose that the evaluation of gait variability may also be addressed with respect to both performance and elemental variances. The overall gait pattern (i.e., the whole-body propulsion expressed as velocity) may be seen as the performance variable. Body propulsion is a product of the ground reaction forces derived from the foot contact with the ground for each step or stride. These ground reaction forces may be modified by the timing and/or the placement of the feet on the ground and by joint torques generated in the body (especially the ankle, knee and hip joint). The impact of these forces from each foot contact may be regarded as elemental variables. The performance variable, as indicated by the overall gait velocity, is modified continuously by adjustments of the elemental variables, as expressed by the velocity of each step or stride.

A variation in the stride-to-stride sum (an unintended deviation in the overall gait velocity, i.e., performance variable) is regarded as “resulting” variance in the present study. A deviation in stride-to-stride differences (when a deviation in one stride is positively compensated within the following stride, i.e., elemental variables) is regarded as “adjusting” variance in the present study. If the resulting variance is a sign of a loss of energy and the adjusting variance is seen as a natural part of movement adjustments, the ratio between the *adjusting* and *resulting* variations may be expected to serve as an indicator of motor performance during gait.

### Objective

This proof of concept study explored the relevance of a new approach to gait variability evaluations based on the existence of a meaningful ratio between the elemental (stride-to-stride difference) and the performance (stride-to-stride sum) variables of gait. The study also investigated the timing of such stride-to-stride correction mechanisms during treadmill walking.

## Methods

### Participants and experimental setup

Twenty young and healthy subjects (13 females and 7 males) participated in the study. The mean age of the study population was 24 years (SD 3.7), and the mean body mass index was 22.9 (SD 2.4). The participants walked on an instrumented treadmill with the speed set at their preferred gait velocity. At first, they walked for 3 min to become accustomed to walking on the treadmill. Then, the gait pattern was recorded for 1 min. After 10 min of rest, this procedure was repeated.

The sensors in the treadmill (FDM-T; Zebris Medical GmbH) provided “electronic footprints” from which the temporal and spatial parameters of the gait could be derived. The pressure platform in the treadmill had 1.4 sensors per cm^2^, and the sampling rate was set to 100 Hz. The raw data were exported to Matlab (Mathworks, R2008a) for offline analysis. Heel strike was identified for each step and a stride was defined as the sequence from one heel strike to the following ipsilateral heel strike. Stride time and stride length were extracted from these data, and the velocity (cm/s) of each stride was calculated (stride velocity = stride length/stride time). The sole stride data (not the step data) were used because the differences between the gait cycles were the focus of the study and because of the possibility of bias in the step-to-step differences caused by asymmetry in the gait pattern.

### Stride-to-stride interaction analysis

The natural gait pattern is not stereotyped but constantly adjusted over time. To ensure a relatively steady gait pattern, any tendency towards deviation in gait velocity during one stride must be corrected at some point within the few following strides. The existing literature suggests that such gait variability can be evaluated using a stride-to-stride comparison [12, 13]. However, the optimal time lag value to be used in the proposed gait variability analysis is unknown. For a stride-to-stride comparison, the smallest possible time lag value is 0.5 stride. We expected meaningful gait corrections to occur within two full gait cycles, considering the added constraints in space and time imposed by walking on a treadmill [26]. Consequently, we set the largest time lag value to 2.

The gait pattern was examined relative to the presence of a stride-to-stride interaction by matching the velocity of one baseline stride (stride_i_) to the velocity of the following stride with four different time lags (stride_i+x_). The subsequent stride would be one of the following:

- The following contralateral stride (stride_i+½_)
- The following ipsilateral stride (stride_i+1_)
- The second following contralateral stride (stride_i+1½_)
- The second following ipsilateral stride (stride_i+2_)

**Fig. 1:**
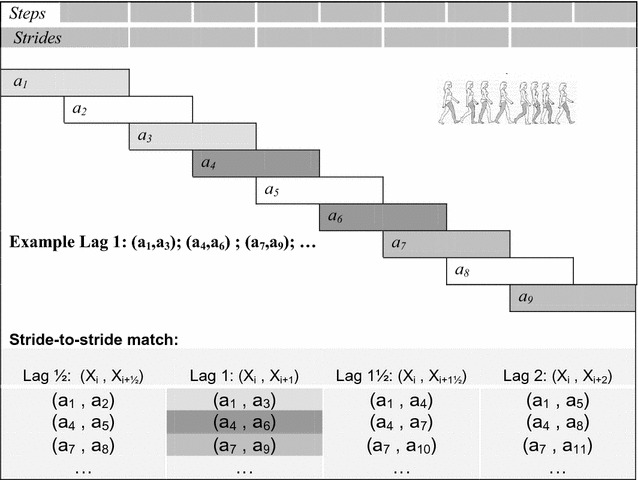
Illustration of the stride-to-stride comparison with different time lags. Three of the lag-1 comparisons are presented in the graphics with *shaded colours*. For each iteration, the next iteration was performed three steps later

For each iteration, the next iteration was performed three steps later (see Fig. 1). This was done to ensure independence between strides and to avoid potential effects due to a bivariate relationship.

**Fig. 2:**
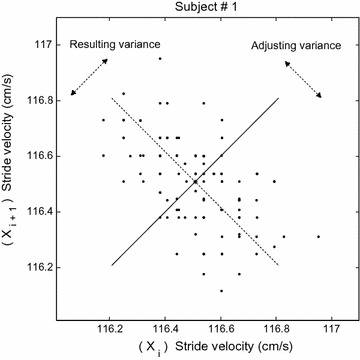
Stride-to-stride relationships. An example of stride velocity (cm/s) plotted for each stride (X_i_) with respect to the following ipsilateral stride (X_i+1_). The *adjusting* variability was evaluated with respect to the *diagonal line* with the positive slope, and the *resulting* variability was evaluated with respect to the *dashed line* with the negative slope

The baseline stride velocity was plotted against the velocity of the subsequent stride in a coordinate system to enable a visual evaluation of the interaction between *adjusting* and *resulting* variances (Fig. 2). The *adjusting* and *resulting* variances were evaluated in the diagonal directions of the plot. This means that the horizontal and vertical axes were rotated 45° anticlockwise for the variance analysis. *Adjusting* variance presents in the direction along a straight line with a negative slope (i.e., relative to the distance from the line with a positive slope travelling through the mean of the data points [X_mean_, Y_mean_]). This variance would reflect the corrections from one stride to another that were considered necessary and relevant adjustments. By contrast, the variance in the orthogonal diagonal direction (relative to the distance from the line with a negative slope) would reflect a variation in gait velocity (*resulting* variance). Large deviations in overall gait velocity are considered nonoptimal.

In a mathematical sense, the *adjusting* variance may also be explained as the variance in the differences in stride-to-stride velocity and the *resulting* variance may be explained as the variance in the sums of the stride-to-stride velocity.

The *adjusting/resulting* variance ratio may therefore simply be calculated as the variation in stride-to-stride differences divided by the variation in the stride-to-stride sums:

$$ \left( {{\text{variance}}\left[ {{\text{stride}}_{\text{i}}  - {\text{stride}}_{{{\text{i}} + {\text{t}}}} } \right]} \right)/\left( {{\text{variance}}\left[ {{\text{stride}}_{\text{i}} + {\text{stride}}_{{{\text{i}} + {\text{t}}}} } \right]} \right) $$

According to the approach used in this study, a positive stride-to-stride interaction exists when the *adjusting* variance is larger than the *resulting* variance. This means that a ratio between the *adjusting* and the *resulting* variance >1.0 indicates the existence of an appropriate stride-to-stride interaction.

### Statistical analysis

The data was analysed in Excel (MS Office 2007) and SPSS (PASW ver. 20). The mean values, the standard deviations and the 95% confidence intervals were reported. The *adjusting/resulting* ratio was evaluated by a one-sample *t* test and confidence intervals (95% CI). A P value <0.01 was regarded as significant.

The consistency between trials 1 and 2 was described by intra-class correlations (ICC_3.1_), averaged differences and typical error (TE). ICCs were interpreted as 0.0–0.4, unacceptable; 0.4–0.6, moderate; 0.6–0.8, substantial; and 0.8–1.0, almost perfect agreement according to Landis and Koch [27]. TE = (SD_diff_/√2), where SD_diff_ is the standard deviation of the individual difference scores between the trials.

## Results

The average gait velocity on the treadmill was 1.2 (SD 0.1) m/s. The average stride velocity was 118.2 (5.7) cm/s with an average individual variance of 0.02 (0.01) and a coefficient of variation of 0.12 (0.03).

The parameters related to the stride-to-stride interaction approach, i.e., *adjusting* and *resulting* variances and the ratio between these, are presented in Table 1.

**Table 1:** Stride-to-stride synergy variability parameters for stride velocity

	*Adjusting* variance	*Resulting* variance	*Adjusting/resulting* variance ratio	CI 95%
Trial 1				
lag_i+½_	0.040 (0.019)	0.042 (0.015)	1.0 (0.4)	(0.8–1.2)
lag_i+1_	0.060 (0.025)	0.022 (0.008)	2.9 (1.4)**	(2.3–3.6)
lag_i+1½_	0.043 (0.018)	0.041 (0.019)	1.2 (0.5)	(1.0–1.4)
lag_i+2_	0.042 (0.017)	0.040 (0.020)	1.2 (0.5)	(0.9–1.4)
Trial 2				
lag_i+½_	0.034 (0.011)	0.045 (0.011)	0.8 (0.3)*	(0.7–0.9)
lag_i+1_	0.055 (0.018)	0.022 (0.006)	2.8 (1.5)**	(2.1–3.4)
lag_i+1½_	0.042 (0.012)	0.035 (0.011)	1.3 (0.4)	(1.0– 1.5)
lag_i+2_	0.038 (0.010)	0.040 (0.011)	1.0 (0.4)	(0.9–1.2)

Variance parameters are presented by mean values (SD), as well as confidence intervals for their ratio, with respect to stride-to-stride comparisons with four different time-lags: lag_i+½_ the following contralateral stride; lag_i+1_ the following ipsilateral stride; lag_i+1½_ the second following contralateral stride; lag_i+2_ the second following ipsilateral stride

* P < 0.01

** P < 0.001

**Fig. 3:**
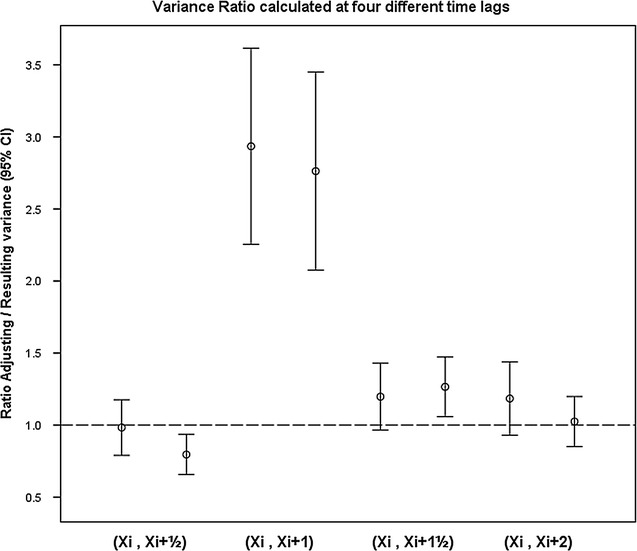
*Adjusting*/*resulting* variance ratios (mean and 95% confidence intervals) are presented in pairs from the two trials. The four sets of ratios represent stride-to-stride comparisons, which were generated with four different time lags (X_i_, X_i+t_)

Figure 3 illustrates the divergence between the different time lags.

In the four time-lag analyses, the ICC_3.1_ values between trial 1 and trial 2 for the *adjusting/resulting* ratio were 0.17, 0.35, 0.43 and 0.06. The corresponding averaged differences were −0.19, −0.17, 0.06 and −0.16, and the TE values were 0.34, 1.30, 0.40 and 0.46.

## Discussion

### Summary

The approach to the gait analysis used in this study evaluated the variation in stride-to-stride velocity differences, i.e., the *adjusting* variability, relative to the variation in stride-to-stride sums, i.e. the *resulting* variability of the gait velocity. A stride-to-stride interaction pattern was observed during treadmill walking. This was reflected in an *adjusting*-*resulting* variance ratio of 2.9 (CI 95% 2.3–3.6) when comparing the velocity of strides to their subsequent ipsilateral strides. According to this analysis, the adjustment of the gait velocity occurred with a time lag of 1 full stride in asymptomatic young adults.

### Stride-to-stride evaluation

The relevance of gait pattern analysis has been illustrated in a study by Hausdorff et al., in which increased stride-to-stride time variability was found to indicate poor balance and a risk for fall among elderly subjects [12]. The method used in the present study evaluated the data from one stride relative to the following stride. This approach is consistent with the approach suggested by Slifkin and Newell [28]. They stated that the standard deviation, a measure of the size of the variability, does not provide a complete picture of variability because other constructs of variability like the structure of the time series also contains important information [28]. Moe-Nilssen et al. analysed the structure of the time series using an autocorrelation coefficient to detect gait deficits [5]. This method demonstrated validity in the discrimination between fit and frail elderly persons [29]. The autocorrelation is, however, sensitive to the number of steps included in the analysis sequence. More sophisticated analyses, including non-linear dynamics, have also proven to be useful in gait analyses [30, 31].

A simple and well-known method for assessing the structure of system output is to view plots of relationships between each data point (*x*_*t*_) and the next data point in a time series, i.e., data embedding (see Fig. 2). The resulting scatter plot depicts the bivariate relationship of (*x*_*t*_) and (*x*_*t*+*1*_), a relationship that can be quantified as the lag-1 autocorrelation [28]. We chose a standard embedding technique to build a state-space reconstruction using original stride velocities and the delayed forthcoming ones. This choice is important because too-low or too-high values may result in state-space vectors that fold onto themselves or become indistinguishable [32]. In the present study, 1 full stride cycle was found to be sufficient to adjust the gait velocity. This shows that our a priori choice for the smallest and largest time lags were sound during treadmill walking.

### A stride-to-stride interaction approach

Latash and colleagues [19] inspired the stride-to-stride interaction analysis used in the present study. Gait variability may include inter-stride corrections (elemental variables) that ensure a more or less steady gait (performance variable). Such a stride-to-stride evaluation of the gait pattern reflects the traditional clinical observations of the flow and rhythm of the gait pattern. In a clinical context, common sense indicates that a deviation in one stride will be corrected during the following strides to preserve good postural control and the flow of the movement. Therefore, the stride-to-stride interaction approach provides an easy-to-comprehend and affordable (in terms of computing time) means to assess variability, which may also be used in online feedback applications.

### Limitations

The following limitations have been discussed as part of the peer review process:

The use of a treadmill is a certain limiting factor since walking on a treadmill does not reflect a natural gait and may not ensure natural gait adjustments [33]. Considering the explorative nature of the study, the ability to collect data for a large number of steps in a controlled environment was important, and the use of a treadmill for data collection allowed for an analysis of stride length and time over many consecutive strides. We believe that the *resulting* variance may be smaller on a treadmill compared to over-ground walking due to the augmented spatial and temporal constraints when walking on a treadmill [26]. Furthermore, reliability issues have been reported for gait variability parameters [34]. The present study can serve only as a proof of concept. As such, it constitutes a first step toward a better understanding of an alternative approach to assess stride-to-stride variability. Future studies should challenge the relevance of this approach during over-ground walking and challenge its convergent validity by addressing different age groups and patient categories.

The present data revealed that a time lag of 1 full stride was sufficient to adjust gait velocity. This was evident in both trial 1 and trial 2, which may indicate some robustness of the finding. However, we also found low ICCs, indicating unacceptable or moderate test–retest agreement [27]. These low ICCS can mostly be attributed to the homogenous study group and the constraints imposed by the use of a treadmill on the gait pattern, resulting in poor prerequisites for a high correlation [35]. The target groups for the clinical gait examinations are often elderly people and patients with motor deficits. Higher ICC values may be expected when evaluating the reliability in these populations.

## Conclusions

In the present study, stride-to-stride interactions were found during treadmill walking. The gait pattern requires ongoing systematic adjustments of the gait velocity. Such adjustments were revealed to occur within one full stride. In conclusion, we suggest that the evaluation of the *adjusting/resulting* variance ratio during gait could be used to examine variability relative to gait assessments. Future studies that test the validity of this approach in over-ground and in different populations walking are warranted.
